# Brewers' Yeast (*Saccharomyces cerevisiae*) Purified Functional Feed Additives Mitigate Soybean Meal-Induced Enteritis in Atlantic Salmon (*Salmo salar*) Parr

**DOI:** 10.1155/anu/8555658

**Published:** 2025-04-10

**Authors:** Taofik A. Momoh, Sheu Gbolahan Odu-Onikosi, Folasade Damilola Amulejoye, Joshua Wilson, Benjamin Eynon, Holger Kühlwein, Victor Kuri, Daniel L. Merrifield

**Affiliations:** ^1^Fish Health and Nutrition Research Group, School of Biological and Marine Sciences, University of Plymouth, Plymouth, UK; ^2^Department of Fisheries and Aquaculture Technology, Olusegun Agagu University of Science and Technology, Okitipupa, Nigeria; ^3^Leiber GmbH, Bramsche, Germany

**Keywords:** *β*-glucan, Atlantic salmon parr, gut health, soybean meal-induced enteritis, yeast cytosolic extracts

## Abstract

Soybean meal (SBM) is commonly used in aquafeeds due to its wide availability, reasonable protein content, and cost-effectiveness. However, high SBM inclusion levels in the diets of carnivorous fish, such as Atlantic salmon (*Salmo salar*), can cause soybean meal-induced enteritis (SBMIE), resulting in compromised gut health, reduced nutrient absorption, and impaired growth. An 8-week study was conducted to evaluate the potential of brewers' yeast-derived functional feed additives (FFAs), specifically yeast cell wall *β*-glucans (P*β*G [purified *β*-glucan]) and yeast cytosolic extracts (YEs), to mitigate the adverse effects of SBMIE in Atlantic salmon parr. Fish were fed diets containing 30% SBM (30-SBM) with either 0.02% *β*-glucan (30-SBM+P*β*G) or YE at 1% (30-SBM+YE1) and 2.5% (30-SBM+YE2.5) inclusion levels and compared against a control diet without SBM (0-SBM). The study assessed growth performance, haematological parameters, distal intestinal morphology, and the distal intestinal gene expression levels of enteritis biomarkers (*casp3b*, *pcna*, and *hsp70*). The results showed that P*β*G and 1% YE supplementation significantly reduced the severity of SBMIE, with improvements in intestinal morphology, including reduced intraepithelial leukocytes (IELs) levels and goblet cell hyperplasia. Intestinal gene expression levels of *casp3b* and *pcna* were significantly downregulated in the P*β*G and YE fed fish relative to the 30-SBM fed fish, indicating reduced apoptosis and more controlled cell proliferation. However, the effects of 2.5% YE supplementation were less pronounced, indicating a dose-dependent response. These findings demonstrate that both P*β*G and YE from 100% *Saccharomyces cerevisiae* can alleviate SBMIE in juvenile Atlantic salmon by supporting gut health and modulating cellular recovery processes.

## 1. Introduction

The juvenile life stage of Atlantic salmon (*Salmo salar*) is critical for growth, development, gut health, and stress resistance. Nutritional interventions during this period can have profound long-term effects on health, immune function, and overall production performance, especially when challenging levels of plant protein ingredients (PPIs) such as soybean meal (SBM) are used in the diet. SBM remains a staple ingredient of the global aquafeed industry due to its reasonable protein content, acceptable amino acid profile, availability, and price competitiveness [1–3]. However, its inclusion in Atlantic salmon diets is limited by the presence of antinutritional factors (ANFs), including saponins, lectins, and trypsin inhibitors, which cause a pathology known as soybean meal-induced enteritis (SBMIE)—an inflammatory condition in the distal intestine (DI) of fish [2–6]. Morphologically, SBMIE is characterized by the shortening of the simple and complex mucosal folds (MFs) with the widening of the lamina propria (LP) and submucosa (SM), shortening of the microvilli of the brush border membrane, increased formation of microvillar vesicles, an elevated number of goblet cells, infiltration of immune cells into the epithelia and partial to complete erosion of the supranuclear vacuoles (SNVs) [1, 7, 8].

Attempts to mitigate SBMIE include the exploration of functional feed additives (FFAs), such as those derived from brewers' yeast (*Saccharomyces cerevisiae*), which have shown promise in improving gut health and modulating the immune system of fish [9–9]. Depending on the method of downstream processing employed to lyse the yeast cells, different purified products with unique biophysical and biochemical properties can be obtained for different applications [10–12]. Traditionally, such efforts have focused on yeast cell wall components, such as *β*-glucans and mannan oligosaccharides (MOSs), which are known to enhance immune responses, improve gut barrier function, and promote beneficial gut microbiota [13–18]. *β*-glucans interact with pattern recognition receptors (PRRs) like Dectin-1 and toll-like receptors (TLRs), modulating cytokine production, and subsequent immune function [12]. However, little is known of the potential of yeast cytosolic extracts (YEs) which differ from cell wall-derived additives and may offer distinct benefits in enhancing gut health and recovery, particularly during the fast-growing life stages of carnivorous species [19].

YE obtained from *S. cerevisiae* contain bioactive compounds such as nucleotides, peptides, amino acids, vitamins, and cofactors that have been shown to support cellular repair, enhance nutrient absorption, and reduce oxidative stress [17, 20]. Nucleotides, for example, are conditionally essential biomolecules that serve as building blocks for DNA and RNA and are involved in several key biological processes including cell proliferation [21]. While fish can synthesize nucleotides endogenously, the demands for nucleotides increase during periods of rapid growth, immune challenges, and intestinal repair, such as those triggered by SBMIE [22]. In addition to supporting cell proliferation, nucleotides, and peptides from YE have been shown to modulate the innate immune response in other fish species such as common carp (*Cyprinus carpio*), Nile tilapia (*Oreochromis niloticus*), red sea bream (*Pagrus major*), pikeperch (*Sander lucioperca*), and among others [19, 20, 23, 24]. More so, other yeast cytosolic compounds such as glutathione and antioxidant peptides have been reported to mitigate oxidative stress in the gut, further enhancing the protective effects on the intestinal epithelium [19, 20].

Given the importance of understanding how different components of yeast affect Atlantic salmon gut health in the presence of high levels of SBM, this study investigated the effects of YE (at two dosages: 1% and 2.5%) and yeast cell wall *β*-glucan (P*β*G [purified *β*-glucan]; at 0.02%) on juvenile Atlantic salmon fed a diet containing 30% SBM. This comparison aims to elucidate how the different yeast FFAs are able to ameliorate SBMIE.

## 2. Methods

The experiment was conducted at the University of Plymouth, United Kingdom. Fish used for this experiment were handled in accordance with the Animals (Scientific Procedures) Act 1986 (ASPA). The experiment was designed and conducted under UK Home Office Project License PP5489845 and in accordance with the guidelines of the University of Plymouth Animal Welfare and Ethical Review Board (AWERB).

### 2.1. Fish and System Management

After an initial quarantine period of 10 days, the fish were transferred to the East Aquarium Unit of the School of Biological and Marine Sciences, University of Plymouth. The aquarium consists of two interconnected recirculating aquaculture systems (RASs) containing a total of 18 × 75 L rectangular tanks. Each RAS is equipped with a swirl filter incorporated with Japanese nylon filter for mechanical filtration, a biological filter unit containing fluidized biological media (Kaldnes K1), and a P16 commercial UV light system. A 12-h light/dark photoperiod was maintained throughout the experiments using a LED AquaRay control system.

The pH, dissolved oxygen, and temperature were monitored daily using a portable reader (Hach HQ40D Multimeter, HACH, Düsseldorf, Germany). An average pH of 6.7 ± 0.3 was maintained by adding sodium bicarbonate (NaHCO_3_) buffer as necessary to make up for acidification from nitrification and fish gaseous exchange. Dissolved oxygen level was kept at 9.3 ± 0.2 mg L^−1^ with additional aeration provided through air stones while water temperature was maintained at 16 ± 0.5°C. Ammonium (Hach Lange LCK 304), nitrite (Hach Lange LCK 341), and nitrate (Hach Lange LCK 340) were monitored on a weekly basis, with average measurements of 0.03 ± 0.02 mg L^−1^, 0.02 ± 0.01 mg L^−1^, and 19.4 ± 5.2 mg L^−1^, respectively. Water exchange of ~1.5% of system volume was conducted daily to control nitrate levels.

### 2.2. Diets Formulation and Experimental Design

All the diets were formulated to meet the known nutritional requirements of Atlantic salmon parr [25] and were fed to the experimental animals for 8 weeks. SBM was included at 30% of the formulation as the positive control treatment (30-SBM) or enteritic model. A nonenteritic model was formulated without dietary SBM as the negative control (0-SBM). In addition, three treatment diets were formulated to include 30% SBM and yeast FFAs as follows: 30-SBM+P*β*G (30% SBM+0.02% Leiber Beta-S), 30-SBM+YE1 (30% SBM+1% Leiber soluble dried yeast extract), and 30-SBM+YE2.5 (30% SBM+2.5% Leiber soluble dried yeast extract) (Table 1). Both yeast FFAs were obtained from Leiber GmbH and derived from 100% brewers' yeast (*S. cerevisiae*).

In terms of the bioactive components, the P*β*G treatment contained highly purified *β-1,3-1*,6-glucan from the cell wall while the YE treatments contained nucleotides and other extracts from the nutrient-dense yeast cytoplasm. Both FFAs were incorporated into the diets following manufacturer's instruction and recommended inclusion levels. All the diets were produced at the University of Plymouth. The ingredients were gently mixed to form a homogenous dough with the addition of warm water using a Hobart food mixer (Hobart Food Equipment, Orton Southgate, Peterborough, United Kingdom, model no: HL1400—10STDA mixer). The homogenous dough was then cold pressed to form 1 mm diameter pellets (PTM system, model P6, Plymouth, United Kingdom). The proximate composition of the diets (Table 1) was determined according to AOAC [26] methods.

At the start of the experiment, a total of 450 salmon parr (ca. 24 g) were randomly assigned into 15 experimental tanks (30 fish per tank) and fed one of the five experimental diets with each treatment replicated in three tanks. Fish were fed a daily average of 1.5% of their biomass spread over three feeding events. Tank biomass was bulk weighed biweekly to adjust feeding rate.

### 2.3. Sampling

At the end of the experiment, all the fish in each tank were bulk weighed to obtain the final biomass per tank. After this, a random sample of five fish were netted from each tank and euthanized following UK Home Office Schedule 1 (SK1) procedures. The fork length and individual weights of the five fish per tank were measured to obtain the Fulton's condition factor (*K*). Thereafter, 100 µL of whole blood was collected from the caudal vein using a 25-gauge needle and 1 mL syringe. From the whole blood of each fish, 4 µL was immediately fixed in 1 mL of Drabkin's reagent for the determination of hemoglobin concentration and 20 µL in 980 µL of Dacies' solution for differential leukocytes count. In addition, blood smears were prepared on glass slides using 5 µL of whole blood and fixed in 100% ethanol for the determination of blood cell counts. DI samples (ca. 5 mm) were excised from each fish for histological analysis and fixed in 10% formalin at 4°C for 48 h then stored in 70% ethanol until processing. Scanning electron microscopy (SEM) samples (~5 mm) were washed in 1% S-carboxymethyl-L-cysteine buffer (pH 7.2) and preserved in 2.5% glutaraldehyde with 0.1 M sodium cacodylate buffer (1:1 v/v, pH 7.2) until processing. Lastly, about 100 mg of DI samples were stored in 1 mL RNA later solution at 4°C for 24 h, then at −80°C until RNA extraction and subsequent gene expression analysis.

### 2.4. Histology

Fixed DI samples were dehydrated in a graded ethanol series using a Leica TP 1020 tissue processor (Leica Biosystems, Nussloch GmbH, Germany) and then embedded in paraffin wax according to standard histological techniques [27]. Ultrathin sections of 4 μm thickness were cut on a Leica RM2235 microtome (Leica Biosystems), dried in an incubator overnight, and thereafter stained manually with Alcian blue–periodic acid schiff (AB/PAS) according to the protocol described by Dimitroglou et al. [28].

Thereafter, slides were immediately mounted with coverslips using DPX (BDH Laboratory Supplies, Poole, UK) and left to dry. Micrographs of the stained samples were captured on a Leica DMD 108 digital microscope at ×400 magnification, and the images were analyzed using Fiji v5.2 (National Institutes of Health, Bethesda, Maryland, USA) software. A semiquantitative scoring system was employed after Urán et al. [29] to evaluate (a) the appearance and length of the MFs, (b) the degree of infiltration of intraepithelial leukocytes (IELs) into the LP and SM, (c) the density of goblet cells, (d) the degree of widening of the LP, and (e) the presence and size of SNV. Each criterion was given a score of 1–5 according to Table 2.

### 2.5. SEM

Fixed DI samples were rinsed twice with 0.1 M sodium cacodylate buffer (pH 7.2) for 15 min and thereafter dehydrated by placing samples in graded ethanol solutions (30%, 50%, 70%, and 90%) for at least 15 min each and then twice in 100% ethanol. This was followed by critical point drying and gold spurting of the dried samples as already described by Merrifield et al. [30]. Images of samples were then taken at 15 kV and ×20,000 magnification using a Jeol JSM 5600 LV electron microscope (Jeol, Tokyo, Japan).

Ten images per sample (*n* = 150 images per treatment) were blind analyzed to prevent bias using FIJI v5.2 (National Institutes of Health, Bethesda, Maryland, USA) software to assess microvilli density according to Merrifield et al. [30]. The images were first converted to 8-bit images and then the foreground (microvilli) was differentiated from the background (space between microvilli) by thresholding the image. The ratio of foreground to background was then calculated to give a density value (as arbitrary units, AUs).

### 2.6. Hematology

Hemoglobin was determined using Drabkin's cyanide-ferricyanide solution (Sigma–Aldrich Ltd., Poole, UK). Blood samples preserved in Drabkin's solution were vortexed and incubated for 5 min before measuring absorbance at 540 µm using a spectrophotometer. The sample hemoglobin levels (g dL^−1^) were determined against a standard curve of porcine hemoglobin (Sigma–Aldrich Ltd., Poole, UK). Leukocytes and erythrocytes counts were determined following the original protocol by Dacie and Lewis [31, 32]. The mixture of blood and Dacies solution was vortexed for 60 s to ensure a homogenous solution. A 5 µL subsample was then aliquoted onto a hemocytometer and a minimum of 500 cells were counted for a statistically valid result. Blood smears for differential leukocytes count were air-dried, fixed in 100% ethanol for 15 min, and stained with May Grunwald–Giemsa stain according to the protocol described in Adeoye et al. [33]. Once dried, the slides were mounted in DPX. Lymphocytes, granulocytes, and monocytes were identified following the descriptions of Rowley (1990). A minimum of 100 cells per sample were counted and the values were expressed as a percentage of the total leukocytes.

### 2.7. Gene Expression Analysis

#### 2.7.1. RNA Extraction and cDNA Synthesis

Total RNA was extracted from samples using TRI reagent (Ambion, Life technologies, UK) following the protocol reported by Rawling et al. [34]. Briefly, ca. 100 mg of intestinal tissue was lysed in 1 mL of TRI reagent and then homogenized for 40 s to completely dissociate nucleoprotein complexes. Thereafter, 200 μL of chloroform was added to the samples and centrifuged at 12,000 × *g* for 15 min at 4°C before washing the upper aqueous (RNA-containing) phase with 500 µL of isopropanol. RNA pellets were washed using 1 mL of 70% ethanol and after drying, dissolved in diethylpyrocarbonate (DEPC) water and stored at −80°C. RNA concentration was analyzed using a Nanodrop spectrophotometer (Thermo Fisher Scientific, Loughborough, United Kingdom), RNA purity was analyzed by measuring the OD 260/280 and 260/230 absorbance ratios, and RNA integrity was confirmed by 1% agarose gel electrophoresis.

#### 2.7.2. Real-Time PCR Assay

Transcribed cDNA from samples were subjected to PCR reactions in duplicates using the SYBR Green method on a QuantStudio 12k Flex Real-time PCR thermal cycler (Applied Biosystems) following the protocol reported by Rawling et al. [34]. The thermal profile for all reactions was 95°C for 10 min, 40 cycles of 95°C for 15 s, and 60°C for 60 s with fluorescence monitoring occurring at the end of each cycle. Quality checks of PCR product included melt curve analysis which showed a single peak for all reactions revealing the absence of nontarget sequence amplification or primer–dimer formations. No amplification products were observed in the negative controls. To standardize the results, GAPDH and *β*-actin were used as reference genes (Table 3) to eliminate variation in mRNA and cDNA quantity and quality in each sample. The calculation of the stability value “M” of the reference genes, efficiency of all primers, and the expression of target genes was conducted following Vandesompele et al. [35]. The primers used in this study were designed and first reported by Pontefract [36] and validated through a melt curve analysis and PCR efficiency test. The calculated PCR efficiency values for each gene are shown in Table 3.

### 2.8. Growth and Feed Utilization

Growth and feed utilization performance as well as Fulton's condition factor were calculated as follows:

  $$\begin{array}{c} \text{Average weight gain }\left(g\right)=\text{Final average weight}\left(g\right)-\text{initial average weight}\left(g\right), \end{array}$$

  $$\begin{array}{c} \text{Percentage weight gain }\left(\text{\% }\right)=\frac{\text{Average weight gain}\left(g\right)}{\text{Initial average weight}\left(g\right)}\times 100\%, \end{array}$$

  $$\begin{array}{c} \text{Feed conversion ratio }\left({\text{g g}}^{-1}\right)=\frac{\text{Feed offered}\left(g\right)}{\text{Weight gain}\left(g\right)}, \end{array}$$

  $$\begin{array}{c} \text{Specific growth rate }\left(\text{\% }BW{\text{day}}^{-1}\right)=\frac{\ln\left(\text{final weight}\right)-\ln\left(\text{initial weight}\right)}{\text{Time}\left(\text{days}\right)}\times 100, \end{array}$$

  $$\begin{array}{c} \text{Protein efficiency ratio }\left({\text{g g}}^{-1}\right)=\frac{\text{Weight gain}\left(g\right)}{\text{Protein intake}\left(g\right)}, \end{array}$$

  $$\begin{array}{c} \text{Fulton's conditon factor }\left(K\right)=\frac{\text{Body weight}\left(g\right)}{{\text{Fork length}}^{3}\left(cm\right)}. \end{array}$$

### 2.9. Statistical Analysis

Growth and nutrient utilization data were analyzed using the Real Statistics Resource Pack for Microsoft Excel while the rest of the statistical analysis was performed using R (R Core Team, 2022). Charts on R-studio were produced using the gg-plot 2 package [37]. Prior to analysis, all percentage data were transformed using arcsine, and Shapiro–Wilk tests were used to test for normality of the data distribution where *p* > 0.05 is regarded as the threshold for normality. Where data were normally distributed, a one-way ANOVA was conducted, and Tukey's post hoc HSD was used to assess differences between treatments. Where data were not normally distributed, Kruskal–Wallis tests were used and followed by Dunn's test for post hoc differences between treatments. Results are presented as mean ± standard deviation and differences were determined to be significant at *p* < 0.05.

## 3. Results

### 3.1. Distal Intestinal Morphology

**Figure 1:**
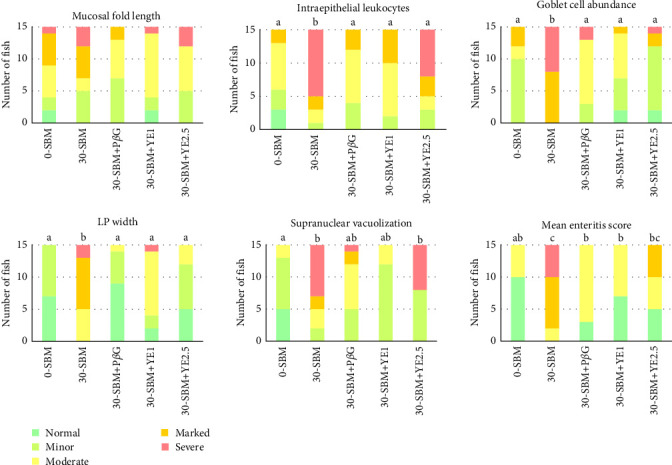
Histopathological changes in the DI of Atlantic salmon parr after 8 weeks of the experiment. An aggregate of the mean weighted score of all five morphological parameters is presented as the mean enteritis score. Treatments with different letters (a–c) above the bars are significantly different (*p* < 0.05). *n* = 15 fish per treatment.

At the end of the 8-week trial, fish in the 30-SBM group showed clear signs of enteritis relative to the 0-SBM group, as indicated by a significant increase in enteritis score for the infiltration of IELs (*p* = 0.002), density of goblet cells (*p* = 0.0007), LP width (*p* < 0.0001), appearance of SNV (*p* = 0.0003), and mean enteritis score (*p* < 0.0001). Only MF length was not significantly different among the treatments. Each of the three yeast-fortified groups (30-SBM+P*β*G, 30-SBM+YE1, and 30-SBM+YE2.5), compared to the 30-SBM group, had significantly lower scores in terms of the infiltration of IELs, goblet cells count, and LP width (*p* < 0.05). Further, there was no significant difference in the size and appearance of SNVs between fish subjected to the 30-SBM diet and those subjected to any of the three yeast-fortified diets. However, fish subjected to both 30-SBM+P*β*G and 30-SBM+YE1 were also statistically similar to the 0-SBM group for this criterion (Figure 1). Finally, the mean enteritis score was significantly higher in the 30-SBM group than all the other treatment groups except the 30-SBM+YE2.5 group.

### 3.2. Microvilli Density

**Figure 2:**
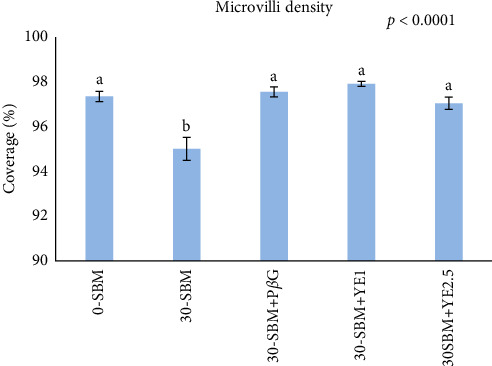
Microvilli density of fish subjected to the different treatments after 8 weeks of the trial. Treatments with different letters (a, b) above the bars are significantly different (*p* < 0.05). *n* = 15 fish per treatment.

**Figure 3:**
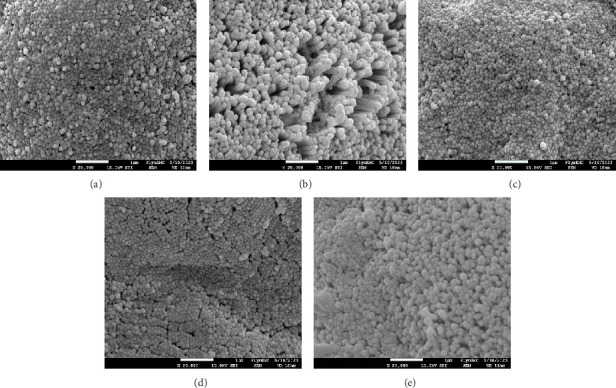
Representative electron micrographs showing the microvilli from the DI of Atlantic salmon parr subjected to the different experimental treatments ((a) 0-SBM, (b) 30-SBM, (c) 30-SBM+P*β*G, (d) 30-SBM+YE1, and (e) 30-SBM+YE2.5). Scale bars = 1 µm. YE, yeast cytosolic extract.

The result from the SEM analysis of the microvilli density on the enterocytes on the apical surfaces of DI mucosa folds is presented in Figure 2. The 30-SBM treatment was significantly different from the 0-SBM and yeast-fortified treatments (30-SBM+P*β*G, 30-SBM+YE1, and 30-SBM+YE2.5) (*p* < 0.0001).  Figure 3 shows representative electron micrographs of the microvilli appearance of the fish subjected to each of the treatments. Particularly, fish fed the 30-SBM diet had a more diffuse microvilli density compared to the rest of the treatments.

### 3.3. Hematology

At the end of the trial, all blood parameters were within the acceptable ranges for Atlantic salmon. There were no significant differences between the treatments for all the parameters except the percentage of monocytes (Table 4).

### 3.4. Transcriptional Response of Enteritis-Related Genes

**Figure 4:**
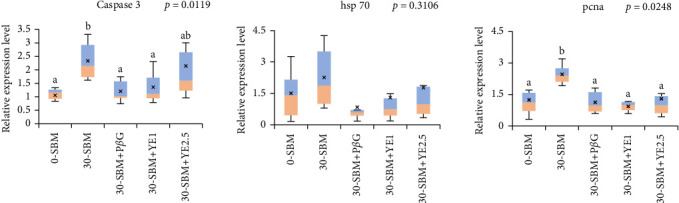
Transcriptional response of enteritis-related genes relative to the reference genes. Treatments with different letters (a, b) above the box plots are significantly different (*p* < 0.05). *n* = 15 fish per treatment.

The transcriptional response of three key enteritis-related genes in the DI of Atlantic salmon parr subjected to the experimental treatments are presented in Figure 4. Compared to the control group (0-SBM), there was a significant upregulation of the relative expressions of *casp3b* (*p* = 0.0018) and *pcna* (*p* = 0.0076) in the 30-SBM group. All the three yeast FFAs (30-SBM+P*β*G, 30-SBM+YE1, and 30-SBM+YE2.5) significantly reduced the expression of *pcna* relative to the 30-SBM group to levels in line with the 0-SBM group. The 30-SBM+P*β*G and 30-SBM+YE1 diets significantly reduced the expression of *casp3b* relative to the 30-SBM group, but this was not the case for the 30-SBM+YE2.5 group. *hsp70* was not differentially expressed by fish in any of the control and treatment groups although its relative expression was numerically higher in the fish fed the 30-SBM diet compared to the others.

### 3.5. Growth Parameters

At the end of the experiment, the largest final average weight was recorded in the 0-SBM group, although this was not significantly different from the other treatments. Consequently, although a similar trend was observed, there were no significant differences in average weight gain, SGR, FCR, and PER (*p* > 0.05) (Table 5).

## 4. Discussion

The objective of the experiment was to assess the ability of yeast-derived FFAs to maintain intestinal homeostasis in Atlantic salmon parr subjected to a challenging level of dietary SBM. A multimarker approach was employed to evaluate the effect of these treatments on intestinal morphology and the transcription of genes related to apoptosis and cellular stress.

### 4.1. Intestinal Morphology

The use of semiquantitative matrices that assign numeric scores to the morphology of the mucosa, using the control treatment as reference, provides a holistic view of the prevalence of SBMIE in fish [2, 5, 6, 38]. The results of this experiment revealed enteritis in the 30-SBM group akin to recent studies on Atlantic salmon where similar levels of SBM were included in the formulation [2, 12, 39, 40]. Particularly, IELs infiltration of the LP and SM, LP width, goblet cell density, and the levels of SNV was significantly different between the 30-SBM group and all the other treatments.

The inclusion of brewers' yeast P*β*G and yeast extracts at 1% (YE1) and 2.5% (YE2.5) resulted in notable improvements in intestinal health when compared to the basal 30-SBM diet. This aligns with previous studies that have highlighted the ability of yeast-derived feed additives to modulate gut microbiota and improve gut morphology in fish under dietary stress. For instance, Gu et al. [15] investigated the effects of 2 g kg^−1^ yeast *β*-glucan supplementation on the gut health of turbot (*Scophthalmus maximus*) fed high levels of SBM. In that study, yeast *β*-glucan supplementation significantly reduced IELs infiltration and goblet cell proliferation, which is consistent with the observed reduction in IELs and goblet cells in the (30-SBM) +P*β*G and +YE groups in the present study. *β*-glucan recognition by pathogen-associated molecular patterns (PAMPs) receptors is known to induce, through the NF-*κ*B pathway, the expression of not only pro-inflammatory but also anti-inflammatory cytokines like IL-6, IL-10, and IL-11 [41]. Similarly, yeast-derived nucleotides supplementation in carnivorous fish diets may increase the expression of the anti-inflammatory cytokine transforming growth factor *β* (TGF-*β*) in the intestine of low fishmeal (FM) fed fish, potentially reducing inflammatory response [19]. While the expression of anti-inflammatory cytokines was not evaluated in the current study, the mechanisms involved in the regulation of inflammatory response by yeast additives may explain the reduced IEL numbers in the yeast fed groups. More so, the significant reduction in goblet cell density in the DI of fish fed the supplemented diets also suggests an important role of the yeast additives in regulating mucosal barrier function. Goblet cells secrete mucus as a protective barrier against pathogens, but an overproduction is often a response to chronic inflammation [42, 43], as observed in the 30-SBM group. The combination of the reduced proliferation of both IELs and goblet cells in the P*β*G and YE—supplemented groups may suggest that these yeast additives conferred beneficial effect on the fish intestinal health by modulating the immune response in the intestine and reducing excessive immune cell infiltration into the epithelium.

Furthermore, P*β*G and YE supplementation in this experiment was seen to counter the excessive swelling and consequent expansion of the LP, characteristic of SBMIE. Studies have highlighted the ability of yeast-derived FFAs to enhance the intestinal barrier function by promoting tight junction integrity and reducing intestinal permeability under nondietary-challenged conditions [44–46]. The observed reduction in the translocation of SBM-induced inflammatory mediators into the SM may further limit the inflammatory response and preserve tissue structure as observed in the yeast-supplemented groups. In addition, there were no significant differences between fish supplemented with either P*β*G or YE at 1% with the 0-SBM group in terms of the appearance of SNV and microvilli density. Yeast additives, particularly those rich in nucleotides may enhance enterocyte proliferation and differentiation, aiding in the recovery of the intestinal epithelium from inflammation-induced damage [17, 19]. However, the lack of significant difference in the appearance of SNV between the 30-SBM group and YE supplementation at 2.5% may suggest a limited ability of the additive to promote tissue repair at high inclusion levels. Previous studies have shown that both *β*-glucans [47] and nucleotide-rich extracts [48] tend to have dose-dependent effects on gut health. According to Pelusio et al. [19], the modulation of PRRs and TLRs by YEs may reduce gene expression of pro-inflammatory cytokines (e.g., TNF-*α*, IL-1*β*) and enhance the secretion of anti-inflammatory cytokines like IL-10 and TGF-*β*, thereby promoting immune homeostasis. As such, supplementation at higher doses may overstimulate the immune system or gut barrier responses, leading to undesirable effects like oxidative stress which could potentially contribute to epithelial stress and damage rather than promote recovery [20]. While this study revealed that YE supplementation at 1% resulted in better morphological indices of the DI than the 2.5% supplementation, a dose-dependent study using graded levels of the YE additive may provide more insight into the optimal level of supplementation.

### 4.2. Expression of Enteritis-Related Genes

The expressions of *casp3b*, *hsp70*, and *pcna* were analyzed to gain insight on the response and recovery of intestinal tissues. While *casp3b* and *pcna* were significantly upregulated in the 30-SBM group compared to the other treatments, the expression of *hsp70* was not significantly different among the treatments. *Casp3b* is a key executor of apoptosis, and its activation is closely linked to the initiation of programmed cell death in response to inflammation, tissue damage, and cellular stress [49–52]. A study by Bakke-McKellep et al. [53] reported significant upregulation in *casp3b* following SBMIE inducement in Atlantic salmon. Similar to their findings, the upregulation of *casp3b* in the 30-SBM group in the current study corroborates the significant loss of supranuclear vacuolization as well as decreased microvilli density observed in the DI morphology of the fish in that group, suggesting increased epithelial cell death. However, supplementation with P*β*G and YE significantly reduced *casp3b* expression, suggesting that both additives mitigated the apoptotic response, potentially by modulating the immune system, and reducing inflammation. Activation of PRRs on macrophages and dendritic cells could induce a shift from a pro-inflammatory to an anti-inflammatory response, thereby reducing the inflammatory milieu, lowering the expression of apoptotic mediators like TNF-*α*, and consequently decreasing *casp3b* activity [54]. The activation of PRRs by yeast *β*-glucans can also inhibit the mRNA expression of the NF-*κ*B pathway, which is central to the production of proinflammatory cytokines. Inhibition of NF-*κ*B reduces inflammation and apoptosis, as NF-*κ*B is known to drive the transcription of proapoptotic genes, such as Fas-ligand (FasL) and *casp3b* [55].

Similar to *casp3b*, the expression of *pcna* was significantly affected by the high level of SBM and the yeast treatments. *pcna* is a marker of cellular proliferation, particularly associated with DNA replication during cell division [56, 57]. In the present study, *pcna* expression was significantly elevated in the 30-SBM group, reflecting compensatory cell proliferation to repair the epithelium in response to tissue damage and inflammation [57]. Supplementation with P*β*G and YE resulted in lower *pcna* expression compared to the 30-SBM group. It could be inferred from this that both yeast additives reduced the need for excessive cell proliferation by ameliorating intestinal damage and maintaining epithelial integrity. According to Grammes et al. [58], the inclusion of 200 g kg^−1^ torula yeast (*Candida utilis*) in the diet of Atlantic salmon parr countered the negative effects of high amounts SBM in the diet, maintaining healthy *pcna* expression levels similar to the control FM diet. Contrastingly, in that same study, the inclusion of brewers' yeast (*S. cerevisiae*) at 200 g kg^−1^ did not significantly counter the enteritic effect of SBM nor reduce the overexpression of *pcna*, suggesting a species effect in the efficacy of yeast products to do so. Other studies indicate that factors such as yeast fraction [34, 44, 59], dosage [13, 47], as well as methods of downstream processing [10–12] may influence the ability of yeast-derived FFAs to confer inflammatory- and immune-regulating properties.

*hsp70* is a molecular chaperone involved in stabilizing unfolded proteins and preventing protein aggregation under stress and is often upregulated during periods of tissue inflammation and cellular stress [8, 59, 60]. Although the difference was not statistically significant, the yeast FFAs reduced the relative expression of *hsp70* in the DI of fish subjected to those treatments likely by modulating oxidative stress levels and stabilizing the gut environment, thereby reducing the need for stress-induced *hsp70* expression [61]. However, the lack of statistically significant changes in *hsp70* expression could also indicate that yeast additives primarily modulate inflammation and cell turnover rather than directly altering cellular stress response pathways [60].

### 4.3. Hematology

In this study, RBC (red blood cell) count, WBC (white blood cell) count, hemoglobin concentration, lymphocyte proportion, neutrophil proportion, and basophil proportion did not differ significantly between treatments. This is consistent with studies where SBM inclusion mainly triggers localized intestinal inflammation without severely impacting systemic blood health in the short-term [33, 62, 63]. The lack of significant changes in these parameters could suggest that SBM-induced enteritis does not cause systemic anaemia or leukopenia in Atlantic salmon parr, at least within the 8-week trial period. However, this may require further investigation with primary focus on the biomechanisms of blood production in fish. Some other studies, for instance on different species of carp, have shown increased monocyte production with *β*-glucan treatment especially under conditions of pathogenic challenge [27, 64, 65]. Notably in this study, there was a significant reduction in monocyte composition in the 30-SBM+P*β*G group compared to the unsupplemented 30-SBM group. However, the reduction in monocyte composition, coupled with no major fluctuations in RBC and WBC counts, may indicate that P*β*G selectively influenced immune cell populations, without affecting other components of the fish's hematopoietic system. Similar immunomodulatory effects of yeast *β*-glucans have been observed in other studies, where they enhance phagocytic activity and modulate cytokine production without further haematological effects [34, 47, 66–68].

### 4.4. Growth Parameters

At the end of the 8-week feeding study, growth and feed utilization parameters were within the acceptable range for Atlantic salmon at this life stage. Attempts to link SBMIE to growth and production parameters in salmonids have yielded conflicting results. Ordinarily, the inclusion of high levels of SBM in fish diet may limit growth through mechanisms such as reduced activities of digestive enzymes (e.g., trypsin; [69]) and reduced digestibility and nutrient utilization [51, 70, 71]. Following the onset of SBMIE, the resulting morphological changes to the DI may further limit nutrient absorption and result in growth retardation [1]. However, similar to this study, several studies have reported the absence of a significant growth retardation in fish displaying enteritis when compared to nonenteritic fish [2, 12, 40, 72, 73]. Although there was some degree of differential growth performance amongst the treatments in this study, ranging between 56.5% (30-SBM) and 64% (0-SBM), the fish biomass only increased by ca. 60% over the study period and was not sufficient to conclusively reveal effects on long-term growth performance. Further, the crude protein level of the 30-SBM diets was slightly higher than the nonenteritis reference diet, SBM-0, which might have masked potential differences in growth performance. While both FFAs in this study showed clear signs of reducing intestinal inflammation and improving gut morphology, these changes might need more time to affect growth performance.

## 5. Conclusions

Given the need to increase global fish production to feed a growing population while reducing the use of FM in diet formulations, the importance of SBM and other PPIs in contemporary and practical diets of Atlantic salmon cannot be overemphasized. Practical diets would typically include lower levels of SBM or rely on more refined, and often more costly, soy derivatives than the levels used in this study. This study employed a diet containing 30% SBM to serve as a challenge model for inducing enteritis, which then enabled the assessment of the efficacy of the yeast-derived FFAs in mitigating SBMIE. This study confirms that feeding a diet with 30% SBM induced enteritis in Atlantic salmon parr, marked by significant inflammatory and structural changes in the DI as well as the significant upregulation of genes related to cellular stress and apoptosis. However, these changes were offset to varying degrees by the inclusion of both yeast FFAs investigated. Particularly, the inclusion of P*β*G at 0.02%, YE at 1% and relatively less successfully, YE at 2.5% improved gut health in the fish subjected to those treatments to a similar level as those in the 0-SBM control group. The implications of these treatment effects over longer trial periods, which include a broader range of yeast additive dosages, remain to be elucidated. Furthermore, the use of integrative bioinformatics approaches to correlate changes in the gene expression with protein abundance and metabolite levels may offer deeper insights into the cellular mechanisms involved in the amelioration of SBMIE in Atlantic salmon parr. Lastly, this study did not include the economic and environmental impact analysis of the different treatments, as such, further research is required to ascertain the cost–benefit relationship as well as the life cycle assessment of the high-SBM yeast-fortified diets, especially under large-scale commercial settings.

## Figures and Tables

**Table 1:** Ingredient and nutrient composition of the experimental diets.

Ingredients (g kg^−1^)	0-SBM	30-SBM	30-SBM+P*β*G	30-SBM+YE1	30-SBM+YE2.5
Soybean meal 48 SKT^a^	0	300	300	300	300
LT fishmeal	100	100	100	100	100
Corn/maize gluten meal	200	265.9	265.7	252.9	233.4
Soy protein concentrate 62	100	50	50	50	50
Sunflower meal	248	20	20	20	20
Wheat gluten meal	118	50	50	50	50
Fish oil	80	80	80	80	80
Sunflower oil 100	88	84	84	84	85
Lysine HCL^b^	20	20	20	20	20
Threonine^b^	10.4	0.5	0.5	10	3.0
Histidine^b^	3.2	6.8	6.8	2.6	10
DL methionine^b^	2.8	3.2	3.2	3.3	3.4
Arginine^b^	20	10	10	10	10
CMC^b^	5	5	5	5	5
Fish premix (0.3%)^c^	5	5	5	5	5
Beta-S^d^	0.0	0.0	0.2	0.00	0.00
Soluble dry yeast extract^d^	0.0	0.0	0.0	10	25
Total	1000	1000	1000	1000	1000

Nutrients (g kg^−1^)	—	—	—	—	—
Dry matter	956	963	965	957	962
Crude protein	470	515	519	512	520
Crude fat	198	202	193	199	188
Ash	46	46	47	46	47

*Note:* All ingredients were sourced from BioMar UK Ltd., except as otherwise stated.

Abbreviations: CMC, sodium carboxymethyl cellulose; YE, yeast cytosolic extract.

^a^Skretting France, Fontaine les Vervins.

^b^Sigma–Aldrich, Poole, UK.

^c^Premier nutrition vitamin/mineral premix (contains 121 g kg^−1^ of calcium, 5.2 g kg^−1^ of phosphorous, 15.6 g kg^−1^ of magnesium, 250 mg kg^−1^ of copper (as cupric sulfate), 7.0 g kg^−1^ of Vit E (as alpha-tocopherol acetate), 1.0 µg kg^−1^ of vit A, 0.1 µg kg^−1^ of Vit D3, and 787 g kg^−1^ ash).

^d^Leiber GmbH.

**Table 2:** Enteritis scoring index [29].

Score	Description	Mucosal folds	Infiltration of IELS	Goblet cells density	LP	SNV appearance
1	Normal	Basal length	Few in SM basal small quantity	Scattered cells	Normal size LP	Basal SNV size
2	Minor	Some shrinkage and bloating	Increased number in SM and some migration into LP	Increased number and sparsely distributed	Increased size of LP	Some size reduction
3	Moderate	Diffused shrinkage and onset of tissue disruption	Increased migration into LP	Diffused number widely spread	Medium size LP	Diffused size reduction
4	Marked	Diffused tissue disruption	Diffused number in LP and SM	Densely grouped cells	Large LP	Onset of extinction
5	Severe	Total tissue disruption	Dense leucocytes in LP and SM	Highly abundant and tightly packed cells	Largest LP	No SNV

Abbreviations: IELs, intraepithelial leukocytes; LP, lamina propria; SM, submucosa; SNV, supranuclear vacuoles.

**Table 3:** Primer details and functions of the genes profiled in qPCR.

Gene	Accession number	Primer sequence (5-3′)	Amplicon size	Annealing temp. (°C)	Efficiency	Function
*gapdh*	AY863148	*Fw*-TGCTGCTTTCACCTCCAAGAA *Rev*-CCATGTACTCCAGGTCGATGAA	75	60	2.00	Reference gene
*β-actin*	NM_001123525.1	*Fw*-ACGGCATCGTCACCAACTG *Rev*-CTCCTCTGGTGCCACTCTCA	83	60	1.99	Reference gene
*casp3b*	DQ008069.1	*Fw*-CCAATGACCAGACTGTGCAA *Rev*-ATGGCTCAGCATCACACACA	101	58	1.97	Apoptosis
*pcna*	XM_014161524.1	*Fw*-CCCTGGTGGTGGAGTACAA *Rev*-TGAAGCCTCCTCGTCAATCT	80	60	1.99	Cell proliferation
*hsp70*	KU885451.1	*Fw*-TCCTGGTGAAGATGAGGGAGAT *Rev*-TAGTGGCCTGTCTCTGTGAATC	108	60	2.00	Cellular stress

**Table 4:** Hematology of fish subjected to the different treatment groups after 56 days of the feeding trial.

Parameters	0-SBM	30-SBM	30-SBM+P*β*G	30-SBM+YE1	30-SBM+YE2.5	*p*-Value
RBC (×10^6^ µL^−1^)	0.7 ± 0.1	0.7 ± 0.1	0.7 ± 0.1	0.6 ± 0.1	0.7 ± 0.1	0.3475
WBC (×10^4^ µL^−1^)	1.0 ± 0.7	0.9 ± 0.5	0.8 ± 0.6	0.7 ± 0.6	0.7 ± 0.2	0.8556
Hb concentration (g dL^−1^)	0.03 ± 0.009	0.04 ± 0.004	0.04 ± 0.008	0.03 ± 0.005	0.04 ± 0.007	0.2780
Lymphocytes (% of WBC)	78 ± 8	78 ± 6	86 ± 6	79 ± 5	78 ± 5	0.1586
Monocytes (% of WBC)	6 ± 3^a^	5 ± 5^a^	2 ± 2^b^	3 ± 2^a^	3 ± 2^a^	0.0349
Neutrophils (% of WBC)	6 ± 4	6 ± 3	6 ± 5	9 ± 4	9 ± 5	0.2206
Basophils (% of WBC)	11 ± 5	11 ± 7	5 ± 4	9 ± 5	9 ± 6	0.3449

*Note:* Values are mean ± standard deviation. Different superscripts signify significant differences between the means of the parameter for fish subjected to the different experimental diets where *p* < 0.05. *n* = 15 fish per treatment.

Abbreviations: Hb, hemoglobin; RBCs, red blood cells; WBCs, white blood cells; YE, yeast cytosolic extract.

**Table 5:** Growth performance and nutrient utilization of fish fed the experimental diets after 56 days of feeding.

Parameters	0-SBM	30-SBM	30-SBM+P*β*G	30-SBM+YE1	30-SBM+YE2.5	*p*-Value
Initial weight (g/fish)	24.4 ± 0.07	24.4 ± 0.10	24.31 ± 0.14	24.27 ± 0.12	24.31 ± 0.08	0.5962
Final weight (g/fish)	40.0 ± 1.57	38.1 ± 1.41	38.7 ± 1.20	38.2 ± 0.75	38.6 ± 1.41	0.4400
AWG (g/fish)	15.6 ± 1.59	13.8 ± 1.48	14.4 ± 1.09	14.0 ± 0.79	14.3 ± 1.35	0.4770
PWG (%)	64.0 ± 6.57	56.5 ± 6.25	59.1 ± 4.24	57.6 ± 3.38	58.6 ± 5.39	0.5030
FCR (g/g)	0.9 ± 0.07	1.0 ± 0.10	1.0 ± 0.07	1.0 ± 0.03	1.0 ± 0.10	0.3580
SGR (%BW/day)	1.4 ± 0.11	1.3 ± 0.11	1.3 ± 0.08	1.3 ± 0.06	1.3 ± 0.10	0.5120
CF (K)	1.1 ± 0.03	1.1 ± 0.03	1.1 ± 0.03	1.1 ± 0.03	1.1 ± 0.03	0.2220
PER (g·growth/g·PI)	1.8 ± 0.17	1.6 ± 0.20	1.6 ± 0.12	1.5 ± 0.02	1.6 ± 0.16	0.2470

*Note:* Values are mean ± standard deviation. There were no significant differences between the means of each parameter for fish fed the different experimental diets. *n* = 3 replicate tanks per treatment for all parameters except CF where *n* = 15 fish per treatment.

Abbreviations: AWG, average weight gain; CF, Fulton's condition factor; FCR, feed conversion ratio; PER, protein efficiency ratio; PWG, percentage weight gain; SGR, specific growth rate; YE, yeast cytosolic extract.

## Data Availability

All the data reported in this article are available upon request from the corresponding authors.
